# Impact of Family Doctor Contract Services on Healthcare Utilization Among Older Adults in China

**DOI:** 10.1093/geroni/igaf122.1609

**Published:** 2025-12-31

**Authors:** Sijiu Wang, Tingyu Lei

**Affiliations:** Tsinghua University, Beijing, Beijing, China (People’s Republic); Tsinghua University, Beijing, Beijing, China (People’s Republic)

## Abstract

As China’s population ages, ensuring access to primary care for older adults is a growing priority. Family doctor contract services have been introduced to strengthen primary care and promote stable patient-provider relationships, yet their effectiveness in improving healthcare access for older adults remains unclear. This study examines the impact of family doctor contract services on healthcare utilization and expenditures among middle-aged and older adults, with a focus on rural-urban disparities. Using data from the sixth (2018) and seventh (2023) Shandong Province Health Service Survey, which employed multi-stage stratified cluster sampling across 20 cities, we analyzed a sample of 36,027 adults aged 45 and older. A panel data approach was used to compare healthcare utilization before and after contract enrollment. Probit and OLS regression models assessed service utilization and expenditures, while instrumental variable analysis, using regional contract signing rates, addressed potential endogeneity. Results indicate that family doctor contracts significantly increased outpatient utilization (6.73%, p < 0.01), with stronger effects in rural areas (13.95%, p < 0.01) than urban areas (2.56%, p < 0.05). Inpatient service use also increased modestly (2.48%, p < 0.05). While outpatient spending initially decreased (10.72%, p < 0.1), this effect became insignificant under IV estimation. Findings underscore the role of family doctors in expanding healthcare access for older adults, particularly in rural regions where primary care is traditionally weaker. Policymakers should prioritize strengthening rural healthcare infrastructure, increasing provider incentives, and addressing disparities to ensure equitable access to primary care as China’s population continues to age.

